# A mouse model for the Sézary syndrome

**DOI:** 10.1186/1756-9966-29-11

**Published:** 2010-02-11

**Authors:** Udo Doebbeling

**Affiliations:** 1 Department of Dermatology, University Hospital Zurich, Zurich, Switzerland

## Abstract

**Background:**

The Sézary syndrome is an aggressive leukemic form of cutaneous T cell lymphoma and there is no cure of this disease. Until now there is no true animal model for Sézary syndrome, by which new drugs against the disease could be tested.

**Methods:**

Immune deficient CB-17 SCID beige mice were injected subcutaneously with HUT78 cells, a cell line, derived from a Sézary syndrome patient. Developing tumors were analyzed by immunohistochemistry.

**Results:**

Injected HUT78 cells formed tumors at the site of injection. In contrast to the Sézary syndrome in man, no malignant cells were observed in the blood of tumor bearing CB-17 SCID beige mice. The tumors appeared 44-62 days after injection and tumor bearing mice survived further 25 - 62 days until they had to be euthanized according to the guidelines of the Swiss animal protection law, since the tumors had reached the maximal allowed size.

**Conclusion:**

Although the mouse model does not exactly match the human disease, it will be suited for tests of new substances for the treatment of the Sézary syndrome. The formation of an isolated tumor on the skin has the advantage that the effect of a potential drug can be directly monitored without the use of invasive methods.

## Background

Cutaneous T cell lymphomas (CTCL) are a class of non-Hodgkin's lymphomas characterized by clonal proliferation of neoplastic T-lymphocytes [1-3]. The two main forms are Mycosis fungoides (MF) and its leukemic counterpart, the Sézary syndrome (SzS). MF remains confined to the skin and often presents with patches and plaques or in more advanced forms with tumors and a generalized erythema (erythroderma). Sometimes MF proceeds to SzS. Sézary Syndrome patients show generalized erythroderma, leukemic T cells in the blood and a reduced life expectancy compared to MF patients with only approximately 30% of patients surviving beyond 5 years after diagnosis. This is probably due to the circulating malignant T cells producing various immunosuppressant molecules such as IL-10, which can lead to down regulation of the immunological tumor surveillance.

Sézary syndrome patients are treated with psoralen and UVA (PUVA) in combination with interferon alpha, locally applied cytostatic substances such as BCNU (1,3-bis(2-chloroethyl)-1-nitrosourea), or low dose methotrexate or radiation therapy [4-6]. These therapies show often complete remission for several months, but the patients relapse. Currently no cure for SzS has been found.

An animal model is a prerequisite for testing newly developed drugs for their efficiency and potential adverse side effects. Animal experiments help to sort out inefficient or harmful compounds that could threaten the health of patients of phase I trials. To study the effects of potential anti-cancer agents often immune deficient mouse strains are used that accept xenotransplants from human tumors or human tumor cell lines. Until now, no true mouse model for the Sézary syndrome is available. Experiments to induce SzS tumors or leukemia in immune deficient mouse strains as athymic nude mice by injecting cells from SzS patients or SzS cell lines have failed. This may be either due to the thin skin of these mice, which may not be ale to deliver the necessary growth factors for the SzS cells, or to the fact that athymic nude mice still possess functional B and NK cells. Here I show that one can induce tumors in CB-17 SCID beige mice, which have no T, B, and NK cells, by injecting cells of the SzS cell line under the skin of these mice.

## Methods

### Cells and cell culture

The cell line Hut78 (Sézary syndrome) was obtained from ECACC. MyLa 2059 and SeAx cells were kindly provided by Keld Kaltoft, University of Aarhus, Denmark. The cell lines were grown in HEPES buffered RPMI 1640 medium supplemented with 2 mM glutamine, 10% fetal calf serum (FCS), 0.25 mg/ml amphotericin B, 100 U penicillin G, 100 U streptomycin and 1 mM pyruvate (all from Invitrogen, the Netherlands).

### Mice and tumor formation

CB-17 beige mice (CB-17/lcr.Cg-Prkdc scid Lyst bg/Crl) were obtained from Taconic (Lille Skensved, Denmark). The mice kept under sterile conditions in the central animal laboratory of the University Hospital Zurich. 3 × 10^6 ^Hut78 cells were injected subcutaneously into the right flank of the mice. Tumors were visible after 5-9 weeks and were grown until the maximal allowed size of 1 cm^3^.

The experiments were performed following the ethic guidelines for animal experiments of the Swiss National Fund and were approved by the Veterinary Authorities of the Kanton of Zurich, Switzerland (license no. 53/2005).

### Immunohistochemistry

Tumors were excised and fixed in formaldehyde and small tumor pieces were embedded in paraffin. Tumor sections were stained by haematoxylin and eosin (HE). For immune histochemistry the slides were probed with antibodies against human CD3 (DAKO, no. A0452, Glostrup, Denmark) and FLIP (Abcam no. 15319). Staining of this antibody was detected using an alkaline phosphatase anti-alkaline phosphatase (APAAP)-immunohistochemistry technique (reagents from DAKO, Glostrup, Denmark).

## Results

### Tumor growth of SzS cells lines on immune deficient CB-17 SCID beige mice

To obtain tumors two groups of seven CB-17 SCID beige immune deficient mice were injected subcutaneously with 3 × 10^6 ^cells of the SzS cell lines HUT78 and SeAx. The injected mice were observed for three months for tumor formation. During this time two tumors were observed in the group that had been injected with HUT78 cells, whereas no tumors were seen in the group that had been injected with SeAx cells. As a positive control two CB-17 SCID beige mice were injected with 3 × 10^6 ^MyLa 2059 cells, which have been shown form tumors on immune deficient athymic nude mice [7,8]. One tumor was observed during the given time span on these animals. Compared to other mouse tumor systems the take on rate of the malignant cells was quite low (28.3% (2/7) for Hut78 cells and 0% (0/7)for SeAx cells).

**Figure 1:**
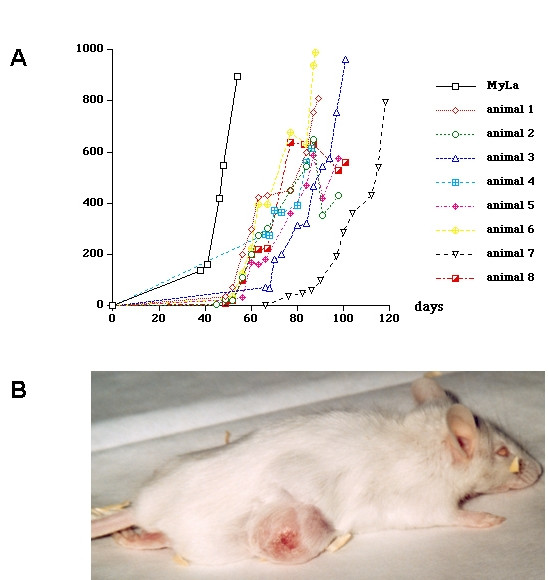
**Tumor formation and tumor growth on CB-17 SCID beige mice injected with 3 × 10^6 ^Hut78 cells**. A) Tumor growth on 8 CB-17 SCID beige mice injected with Hut78 cells (animal 1-8). MyLa indicates a control mouse that had been injected with the same number of MyLa 2059 cells. The tumor volume is indicated by the y axis (in mm^3^). The number of days after the injection is indicated by the x axis. B) Clinical picture of a tumor bearing CB-17 SCID beige mouse injected with Hut78 cells.

Since malignant cells derived from tumors that had already grown on mice are more effective in tumor formation, cells derived from these two tumors were cultured in vitro and 3 × 10^6 ^cells of the culture were injected again subcutaneously into 8 further CB-17 SCID beige mice. This time the formation of 6 tumors was observed corresponding to a take on rate of 75% (6/8). The growth of the individual tumors differed markedly (Figure. 1A). They appeared 5 - 9 weeks after injection. 5 tumors grew continuously and three tumors showed a transient reduction of tumor volume, which was due to the formation of a necrotic area in the center and involution of the central area of the tumor. The growth of the tumor did not cause hair loss in the tumor area and the area had to shaved make the tumors better visible. A clinical picture of a tumor bearing mouse is given in Figure 1B.

The blood of human SzS patients contains malignant T cells. Since the blood of CB-17 SCID beige mice contains no mature lymphocytes they should be easily detected. However, no malignant SzS cells were detected in the blood of the tumor bearing animals, indicating that the malignant human T cells cannot grow in the blood of CB-17 SCID beige mice. The inspection of the inner organs of the tumor bearing mice showed no signs of metastasis formation.

### Morphology of the SzS tumors on CB-17 SCID beige mice

**Figure 2:**
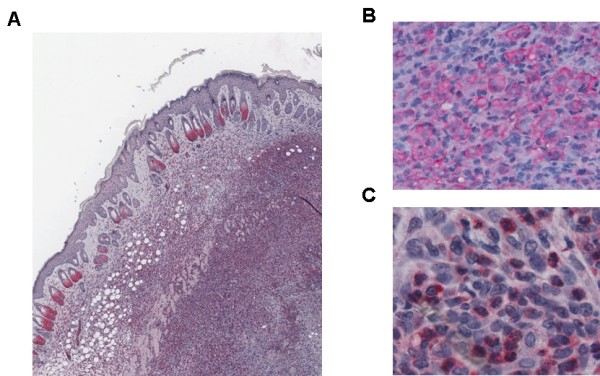
**Morphology of an excised tumor from CB-17 SCID beige mice**. A) Overview. The center of the tumor with necrotic cells is on the bottom on the right side of the figure. The area of living tumor cells can be recognized by the staining with the FLIP antibody. Tumor associated blood vessels appear as white holes. Tumor cells infiltrate the dermis and the epidermis is still intact. Note that the cells at the bottoms of the hair follicles also stain strongly with the FLIP antibody. B) Presence of malignant T cells in the tumor area proven by staining with a CD3 antibody. C) FLIP antibody staining of granulocytes. The FLIP staining cells show the typical segmented nuclei of granulocytes. The original magnifications of the figures 2A, 2B, and 2C were 5×, 20×, and 50× respectively.

The inspection of excised tumors under the microscope, showed that larger tumors contained a necrotic inner center that was covered by zone of living cells. These cells were surrounded by areas that contained atypical blood vessels (Figure 2A), which had mostly only an incomplete endothelium. The tumors consisted of two populations of cells. One population consisted of malignant T cells with large spongiform nuclei. Their identity as malignant T cells (Hut78 cells) was confirmed by staining with an antibody against CD3 (Figure 2B). The Hut78 cells in the tumor appeared as plasma rich malignant T cells, whose plasma membrane stained strongly by the CD3 antibody, confirming the presence of the T cell receptor on these cells. Malignant T cells also infiltrated the dermis and epidermis and caused in some tumors the formation of a visible necrotic area in the center of the tumor.

The other cell population consisted of tumor infiltrating granulocytes, which were easily identified by their segmented and more condensed nuclei (Figure 2c). The granulocytes reacted strongly with an antibody against the anti-apoptotic FLIP protein, whereas the Hut78 only weakly stained with this antibody.

## Discussion

Subcutaneous injection of malignant SzS cells under the skin of CB-17 SCID beige mice led to the formation of isolated tumors at the sites of injection. In contrast to the Sézary syndrome in man, no leukemic T cells were detected in the blood of the injected mice. No metastases were observed.

In contrast to other malignancies, it has been difficult to establish mouse models for CTCLs as mycosis fungoides and the Sézary syndrome [7-10]. For MF a mouse model has been developed [7] that has already been used for pharmaceutical studies [8], however a true model for SzS was still missing. It has been shown that grafts from SzS patients can survive on CB-17 SCID beige mice [9], but these experiments have never been repeated. Successful experiments with grafts from SzS patients and athymic nude mice have not yet been reported. Thus CB-17 SCID beige mice seem to be better hosts for sensitive tumor cells.

Recently Ito et al. [10] reported that they obtained tumors by injecting cells of the SzS cell line HH under the skin of immune deficient NOD/Shi-scid, IL-2Rgamma(null) mice. The injected cells induced extremely fast growing tumors that reached a size of 1 - 3 cm^3 ^within 10-15 days and also infested the liver within this time. This behaviour is in total contrast to the slow growth of SzS tumors and does not represent the pathobiology of SzS and MF. The cells were only characterized by CD30 staining, an antigen that is only expressed by a minority of MF and SzS tumors, but that is indicative for anaplastic large cell lymphoma (ALCL) cells, which can indeed induce fast growing tumors in immune deficient mice.

It is supposed that MF and SzS cells depend on several growth factors that have to be delivered by the host skin or blood [11-13]. These and other still unknown growth factors in turn activate different signalling pathways that stimulate the expression of survival and growth promoting genes as bcl-2 and c-myc [14-16]. Since immune deficient mice lack functional lymphocytes they are unable to deliver growth factors that are produced by these cells. The lack of these growth factors could be an explanation why "Sézary cells" cannot grow in the blood of CB-17 SCID beige mice. It has also to be taken in account that sometimes a murine growth factor cannot substitute the homologous human growth factor, as the differences in the amino acid sequence is too big, so that a murine cytokine cannot bind to the homologous human receptor. In contrast to HUT78 cells, the injection of SeAx cells under the skin of CB-17 SCID beige mice did not induce tumors. The reason for this is unclear. The HUT78 cell line has been established before more than 30 years and there is evidence that HUT78 cells have become independent of several growth factors [8,14] during their long propagation time in vitro. The SeAx cell line has been established approximately 15 years later and it may still depend of additional growth factors that can not be supplied by a murine host, precluding its growth on immune deficient mice.

## Conclusion

Here I report a mouse system for the Sézary syndrome that is reproducible and reliable. Although this mouse model does not exactly match the human disease, since no malignant T cells were found in the blood, it will allow testing new substances for the treatment of the Sézary syndrome. The formation of an isolated tumor on the skin has the advantage that the effect of a potential drug can be directly monitored without the use of invasive methods. Besides oral and intravenous applications one can also test the directly the effect of a locally applied substance.

## Competing interests

The author declares that he has no competing interests.

## Authors' contributions

All mouse experiments were done by UD. The tumors were isolated and minced by UD and passed to the histology lab. The author read and approved the final manuscript.
